# Immunomodulatory properties of human dental pulp stromal cells: the role of IL-6/JAK/STAT3 pathway and PD-L1

**DOI:** 10.3389/fimmu.2026.1713704

**Published:** 2026-01-30

**Authors:** Rosanna Di Tinco, Alessandra Pisciotta, Giulia Bertani, Giulia Orlandi, Laura Bertoni, Elisa Pignatti, Martina Bonacini, Alessandro Rossi, Anke J. Roelofs, Stefania Croci, Cosimo De Bari, Carlo Salvarani, Gianluca Carnevale

**Affiliations:** 1 Department of Surgery, Medicine Dentistry and Morphological Sciences with Interest in Transplant, Oncology, and Regenerative Medicine, University of Modena and Reggio Emilia, Modena, Italy; 2 Clinical Immunology, Allergy and Advanced Biotechnologies Unit, Azienda Unità Sanitaria Locale-Istituto di Ricovero e Cura a Carattere Scientifico (IRCCS) di Reggio Emilia, Reggio Emilia, Italy; 3 Rheumatology Research Group, Institute of Genetics and Cancer, University of Edinburgh, Edinburgh, United Kingdom; 4 Rheumatology Unit, Azienda Unità Sanitaria Locale-Istituto di Ricovero e Cura a Carattere Scientifico (IRCCS) di Reggio Emilia, Reggio Emilia, Italy

**Keywords:** hDPSCs, IL-6 pathway, immunomodulation, inflammation, PD1/PD-L1 pathway

## Abstract

**Introduction:**

Human Dental Pulp Stromal Cells (hDPSCs) of neural-ectodermal origin hold immunomodulatory properties which make them a source for MSC-based therapies for the treatment of autoimmune diseases.

**Methods:**

In this study hDPSCs were exposed to inflammatory conditions mimicked by co-culture with Peripheral Blood Mononuclear Cells activated with anti-CD3/CD28 (aPBMCs) or treatment with Conditioned Medium (CM) from aPBMCs with or without the addition of a specific IL6 receptor (IL6R) inhibitor. To assess IL-6 effects on PD-L1 expression, hDPSCs were treated with IL-6/sIL6R complex and the activation of IL-6 trans-signalling was investigated. The functional role of IL-6 in modulating PD-L1 protein stability was further confirmed by treating hDPSCs with a proteasome inhibitor.

**Results:**

Our results highlighted that hDPSCs exposed to different inflammatory conditions activate Fas/FasL and PD1/PD-L1 pathways. Moreover, hDPSCs modulate inflammatory cytokines release of aPBMCs from Rheumatoid Arthritis (RA) patients. However, the inflammatory milieu induced the upregulation of IL-6 by hDPSCs, which was demonstrated to be strongly correlated to PD-L1 expression, suggesting its involvement in supporting their immunoregulation. Our data demonstrated that the activation of the IL6/JAK/STAT3 trans-signaling pathway in hDPSCs through stimulation with the IL6/sIL6R complex leads to an increase in PD-L1 protein levels, but not PD-L2, via proteasome inhibition.

**Conclusion:**

Our study demonstrates the activation of the IL-6/PD-L1 axis in response to inflammatory conditions and underscores its potential significance in autoimmune diseases since a dysfunction of this mechanism could lead to the onset and progression of chronic inflammatory disorders.

## Introduction

Human Dental Pulp Stromal Cells (hDPSCs) arise from migrating neural crest progenitor cells and contribute to tissue regeneration and homeostasis by exerting immunomodulatory properties (1–5). These immunosuppressive abilities are not constitutive but are strictly dependent on the establishment of an inflammatory microenvironment (2, 6, 7). Previous studies by our research group have demonstrated that hDPSCs activate immunoregulatory mechanisms exclusively when co-cultured with CD3/CD28-activated peripheral blood mononuclear cells (aPBMCs), while resting PBMCs (rPBMCs) fail to elicit such effects. Likewise, the biological behavior of PBMCs obtained from patients with immune-mediated diseases was modulated by co-cultured hDPSCs (2, 6). The activation of these immunomodulatory properties is driven by different direct and indirect mechanisms. Soluble factors such as indoleamine 2,3-dioxygenase (IDO), transforming growth factor-β (TGF-β) and interferon (IFN) γ are recognized contributors to these processes (8–11). Additionally, direct mechanisms involving Fas/FasL and PD1/PD-L1 pathways have been shown to play pivotal roles in mediating hDPSC-driven immune regulation under inflammatory conditions (2, 7, 12). In this context, the involvement of interleukin-6 (IL-6) has emerged as a compelling area of investigation. Our prior findings revealed that exposure of hDPSCs to aPBMCs upregulates IL-6 levels, suggesting a potential role for this cytokine in the immunomodulatory functions of hDPSCs (2, 6). Interestingly, IL-6, while traditionally recognized as a pro-inflammatory mediator (13), has been recently identified as a key regulator of the immunosuppressive properties of mesenchymal stromal cells (MSCs) (14–18). For example, IL-6-silenced bone marrow-derived MSCs (BM-MSCs) exhibit reduced immunosuppressive capacity (15), whereas IL-6 overexpression enhances these properties (18). The pleiotropic effects of IL-6 are mediated by two different pathways, the classic- and trans-signaling. The first one is activated upon IL-6 binding to IL-6Rα on the cell surface, allowing its interaction with the membrane‐spanning protein IL-6 receptor subunit‐β (gp130). The latter is characterized by IL-6 binding to a secreted form of the IL-6R (sIL6R), followed by the interaction of the IL-6/sIL6R complex with gp130. IL‐6 signaling, via either the classic or trans‐ signalling pathways, involves the engagement of gp130 which leads to activation of the JAK/STAT3 pathway, mediating the intracellular signal transduction (13). Despite these insights, the precise mechanisms linking IL-6 signaling to the immunomodulatory profile of hDPSCs remain largely unexplored. A recent study by Na et al., demonstrated that the IL-6 signaling pathway contributed critically to the immunomodulatory mechanism of human decidua-derived mesenchymal stromal cells (DSCs) by inducing the expression of PD-L1 and PD-L2 immune checkpoints (14). Here, we show how IL-6 contributes specifically to the expression of PD-L1 but not PD-L2, supporting the immune regulatory phenotype of hDPSCs. In particular, this study aimed to investigate the role of IL-6 in the modulation of PD-L1 expression in hDPSCs under inflammatory conditions. We provide novel insights into the activation of the IL-6/JAK/STAT3 pathway and its role in enhancing PD-L1 protein stability, which in turn promotes the immunosuppressive profile of hDPSCs.

## Materials and methods

### Human tissue collection

Whole blood samples were collected from healthy subjects (n=3, age 27–50 years, 1 male and 2 females) and patients with Rheumatoid Arthritis (RA) in the active phase of the disease with insufficient response to conventional synthetic disease-modifying antirheumatic drugs (csDMARDs) (n=5, age 30–74 years, 1 male and 4 females). All samples were obtained at Azienda Unità Sanitaria Locale-IRCCS, Reggio Emilia (Italy). The study was approved by the local ethics committee (“Comitato Etico dell’Area Vasta Emilia Nord,” protocol number 2021/0019473, study 1421/2020/TESS/AUSLRE) and performed in compliance with the Declaration of Helsinki. Written informed consent was obtained from all donors.

### Cell isolation and culture

Human DPSCs were purchased from CTIBiotech (n=3; age 13–15 years, 1 male and 2 females; Meyzieu, Lyon, France) and expanded in standard culture medium (α-MEM with 10% FBS, 2 mM L-glutamine, 100 U/ml penicillin, and 100 mg/ml streptomycin, all from Sigma Aldrich, St. Louis, MO, United States) for immunoselection against STRO-1 and c-kit by using a MACS separation kit (Miltenyi Biotec, Bergisch Gladbach, Germany) as previously described (2). The following primary antibodies were used: mouse IgM anti-STRO-1 and rabbit IgG anti-c-kit (Santa Cruz Biotechnology, Dallas, TX, United States), followed by staining with the magnetically labelled secondary antibodies anti-mouse IgM and anti-rabbit IgG (Miltenyi Biotec). The immune-selected hDPSCs were routinely cultured in standard culture medium at 37°C under 5% humidified CO_2_ and characterized for their mesenchymal immune phenotype by FACS analyses (**Supplementary Figure S1**). For all experiments, stromal cells were used between P3 and P7. PBMCs were isolated from human whole blood samples by using Histopaque-1077 density gradient centrifugation (Sigma Aldrich) according to manufacturer’s instructions (2). In order to mimic the inflammatory microenvironment, PBMCs suspended in standard culture medium (RPMI 1640 medium with 10% FBS, 2 mM L-glutamine, 100 U/ml penicillin, 100 mg/ml streptomycin) were activated with anti-CD3/CD28 beads (1:1 cell:bead ratio, dynabeads™ human T-activator CD3/CD28; Gibco, Thermo Fisher Scientific). aPBMCs were either co-cultured with hDPSCs or kept alone under stimulation for 48 hours. The supernatant enriched with the released soluble factors (named as conditioned media, CM) was collected by centrifuging aPBMCs and stored at –80°C.

### Exposure of hDPSCs to *in vitro* models of inflammation

hDPSCs were seeded at a density of 15,000 cells/cm^2^ in RPMI 1640 medium supplemented with 10% FBS, 2 mM glutamine, 100 units/ml penicillin and 100 mg/ml streptomycin and allowed to adhere overnight. The following day, aPBMCs were added to the hDPSCs culture either directly or using a transwell culture system (0.4 μm transwell inserts; Corning) at a 1:2, 1:5, or 1:10 ratio of hDPSCs to aPBMCs. Alternatively, hDPSCs were exposed to CM diluted with fresh medium (1:1 dilution), in the presence or absence of 20, 50 or 100 µg/ml of the IL6R inhibitor, Tocilizumab (Hoffmann-La Roche, Basel, Switzerland). After up to 72 h, cells were harvested and collected for analysis.

### Activation of IL-6 trans-signalling pathway

To check expression of IL-6Rα and gp130 by hDPSCs, flow cytometry analysis was carried out by using the antibodies reported in **Supplementary Table S1**. To activate IL-6 signalling, hDPSCs were seeded at 20,000 cells/cm^2^ in their standard culture medium (10% FBS) and allowed to attach for 24 hours. Then, cells were cultured overnight in reduced-serum culture medium (1% FBS) and the following day stimulated with 10, 60, 120 ng/ml of IL-6/sIL6R complex (RnD Systems, Minneapolis, MN, USA) for up to 24 hours.

### Quantitative RT-PCR analysis

Total RNA was extracted from hDPSCs using TRIZOL reagent (Invitrogen, Thermo Fisher Scientific) following standard protocols. RNA was quantified using a NanoDrop 2000 spectrophotometer (Thermo Fisher Scientific), and 500ng of total RNA was reverse transcribed into complementary DNA using the Applied Biosystems™ High-Capacity cDNA Reverse Transcription Kit (Thermo Fisher Scientific), following the manufacturer’s instructions. Quantitative PCR was carried out in triplicate with QuantStudio™ 3 Real-Time PCR System using PowerTrack™ SYBR Green Master Mix (Thermo Fisher Scientific). Amplification of a single product of correct size was validated by melting curve analysis. Relative gene expression was quantified by ΔΔCt or standard curve method and normalised to expression of the housekeeping gene RPLP0. The oligonucleotide primer sequences are reported in **Supplementary Table S2**.

### Protein extraction and immunoprecipitation

Cells were lysed in RIPA buffer (0.1% sodium dodecyl sulphate (w/v), 0.5% sodium deoxycholate (w/v), 1% (v/v) Igepal, 2% (v/v) protease inhibitor cocktail (Sigma Aldrich, P8340) and 1% (v/v) each of phosphatase inhibitor cocktails (Sigma Aldrich, P0044 and P5726) in PBS, and extracted protein was quantified via bicinchoninic acid (BCA) assay (Thermo Fisher Scientific). Immunoprecipitation assay for gp130 and IL-6 proteins was performed by using the Immunoprecipitation Starter Pack (GE Healthcare, Little Chalfont, Buckinghamshire, UK) according to the manufacturers’ instructions. Four hundred μg of cell lysates, previously pre-cleared with 30 μl of protein A/G for 2h at 4°C under stirring, were incubated with 4.4 μg of rabbit polyclonal anti-human gp130 antibody (Abcam, Cambridge, UK) overnight at 4°C under agitation. Then, 80 μl of protein A/G, previously equilibrated in the same extraction buffer, were added and incubated overnight at 4°C under stirring. After three washing steps, the bound protein was detached from the resin with the SDS loading buffer and recovered by centrifugation. Extracts immunoprecipitated using a rabbit monoclonal (DA1E) IgG XP^®^ Isotype Control antibody (Cell Signalling Technology, Danvers, MA, USA) were used as negative control.

### Western blotting

Western blot analyses were performed on 25 µg of total protein, or immunoprecipitated proteins. Proteins were separated by SDS-polyacrylamide gel electrophoresis. After semi-dry transfer to nitrocellulose membrane (Bio-Rad Laboratories, Hercules, CA, USA), blots were probed with antibodies listed in **Supplementary Table S3**. Antibody binding was detected using Clarity TM Western ECL Substrate (Bio-Rad), using a Chemidoc Imaging system (Bio-Rad). Fiji ImageJ software was used to perform densitometry analysis as previously described (2).

### Quantification of cytokines in culture supernatants

Eighteen cytokines related to Th lymphocyte activation were quantified in culture supernatants of rPBMCs, aPBMCs, hDPSCs cultured alone and aPBMCs/hDPSCs co-culture for 48 hours by using the Procarta Plex Th1, Th2, Th9, Th17, Th22, Treg cytokine panel (eBioscience, Thermo Fisher Scientific) following the manufacturer’s instructions. Thawed supernatants were centrifuged at 10,000 × g for 1 min at 4°C prior to plating. Eight serial dilutions of cytokine standards plus blank (RPMI + 10% FBS) were included. Data were obtained with the Bio-Plex MAGPIXTM multiplex reader and analyzed with the Bio-PlexTM Manager software. Standard curves were calculated with the five-parameter logistic equation regression method.

### Confocal immunofluorescence analyses

Cells were fixed in 4% paraformaldehyde (PFA), and permeabilized with 0.1% Triton-X 100 or ice-cold 100% methanol when necessary. Then, immunofluorescence analyses were carried out following standard protocols (2). In brief, samples were blocked using Image-IT™ FX (Invitrogen, Thermo Fisher Scientific) for 30 minutes at room temperature and then incubated with antibodies diluted in PBS containing 1% of BSA, as reported in **Supplementary Table S4**. Nuclei were counterstained using 1 μg/ml 4’,6-Diamidino-2-Phenylindole Dihydrochloride (DAPI). A Nikon A1 confocal laser-scanning microscope was used for image acquisition. Serial optical sections were processed with ImageJ software to obtain three-dimensional projections, and image rendering was performed using Adobe Photoshop Software, as formerly described (2).

### Statistical analysis

All experiments were carried out in triplicate by using individual human donors or independent experiments. Statistical analyses were performed using GraphPad Prism v9 software. Tests used to determine statistical significance (p<0.05) are described in figure legends. Pearson’s test was used to perform correlation analysis.

## Results

### Cell-cell contact activated immunomodulatory mechanisms in hDPSCs exposed to inflammation

**Figure 1:**
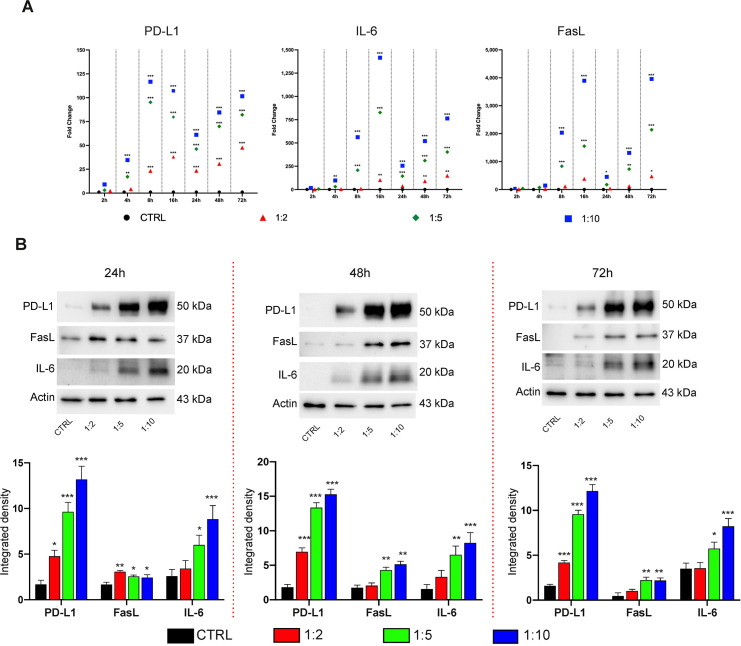
Induction of PD-L1, IL-6 and FasL expression in hDPSCs after direct exposure to inflammation. **(A)** Real Time PCR analyses of PD-L1, IL-6 and FasL in hDPSCs after direct co-culture with aPBMCs at different timepoints (2, 4, 8, 16, 24, 48, 72 hours). Different shapes are colour-coded to identify different hDPSCs-aPBMCs co-culture ratios (1:2, 1:5, 1:10). Data are indicated as mean values of independent experiments using cells from different donors (n=3). **(B)** Western blot analyses showing PD-L1, FasL and IL-6 protein expression in hDPSCs after 24, 48, and 72 hours of direct co-culture with aPBMCs (hDPSCs-aPBMCs; 1:2, 1:5, 1:10 ratios). Histograms report the related densitometric analyses indicated as mean values ± SD (n=3 independent experiments). Original blots are presented in **Supplementary Figure S2A**. In all cases, P values indicate statistical significance obtained by using one-way ANOVA with Tukey’s *post-hoc* test performed within each timepoint (*P<0.05, **P<0.01, ***P<0.001 vs control group).

**Figure 2:**
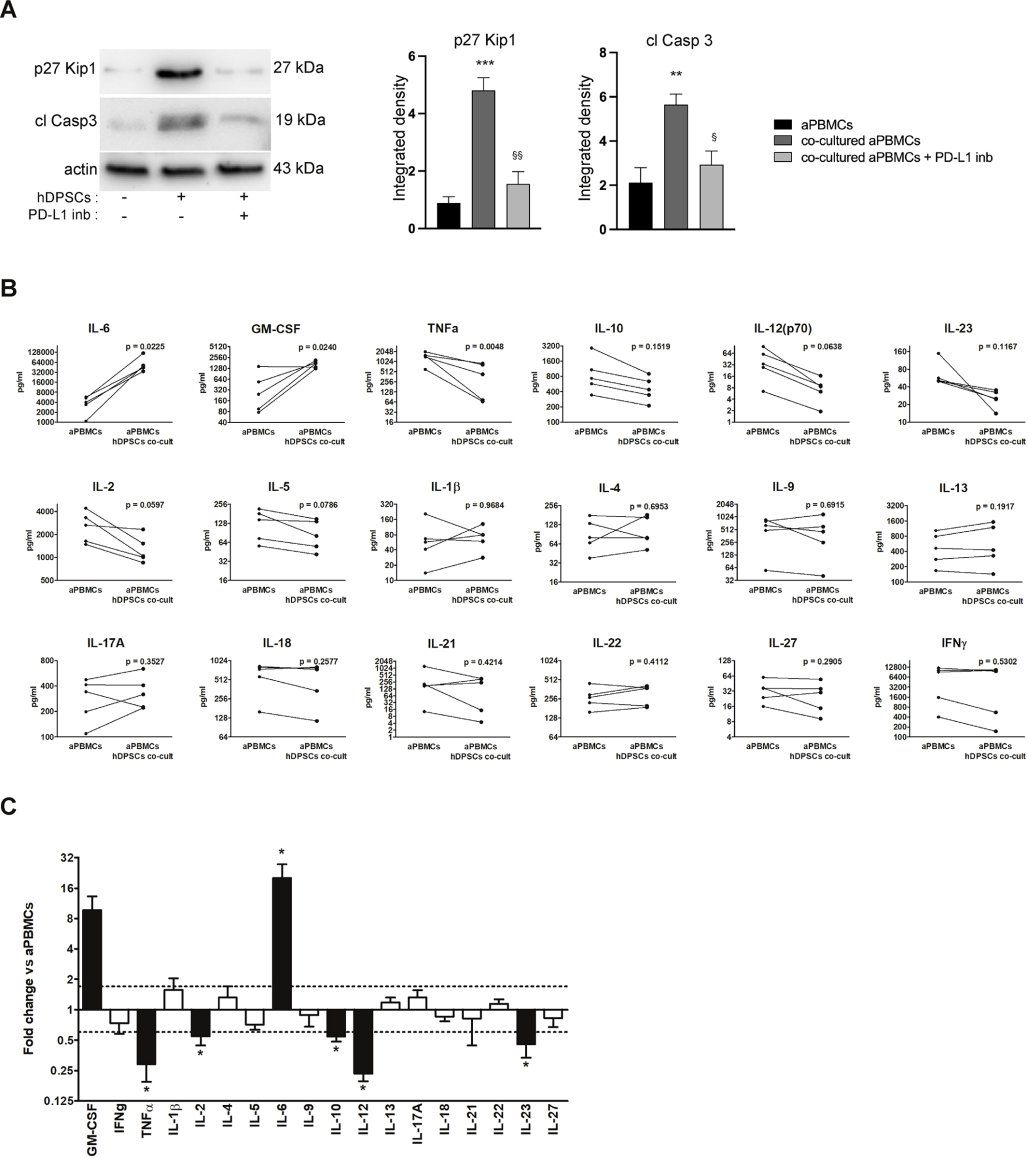
Immunosuppressive effects of PD-L1 on activated immune cells. **(A)** Expression levels of p27 Kip1 and cleaved caspase 3 in aPBMCs cultured either alone or in co-culture with hDPSCs, with or without the PD-L1 inhibitor (10 μg/ml). Histograms show the corresponding densitometric analysis presented as mean values ± SD of three independent experiments using PBMCs from healthy donors (n=3). Original blots are provided in **Supplementary Figure S2B**. Statistical analyses were performed using one-way ANOVA followed by Tukey’s *post-hoc* test (**P<0.01, ***P<0.001 vs aPBMCs, ^§^P<0.05, ^§§^P<0.01 vs co-cultured aPBMCs). **(B)** Concentrations (pg/ml) of 18 cytokines in culture supernatants from aPBMCs of RA patients (n=5) with and without co-culture with hDPSCs (aPBMC and aPBMCs hDPSCs co-cult) were compared using paired t test. **(C)** Cytokine concentration ratios in supernatants from RA aPBMCs co-cultured with hDPSCs versus RA aPBMCs cultured alone were calculated and analyzed using a one-sample t-test, with a reference value set to 1. Fold changes >│1.5│ were considered biologically significant, and p-values < 0.05 were considered statistically significant *P<0.05.

The ability of hDPSCs to interact with inflammatory cells was investigated by directly co-culturing them with aPBMCs, with a specific focus on the expression of the immune checkpoints PD-L1 and FasL. IL-6 expression levels were also evaluated in hDPSCs. Following co-culture, hDPSCs upregulated PD-L1, IL-6 and FasL mRNA levels, which increased proportionally with higher co-culture ratios and over time compared to the control group (*P<0.05, **P<0.01, ***P<0.001 vs control groups; **Figure 1A**; raw data and SD in **Supplementary Table S5**). Notably, we observed an initial up-regulation of all three markers at early time points, reaching a peak at 16 hours of co-culture, followed by a partial down-regulation at 24 hours, and a subsequent increase at later time points (48–72 hours). This trend might be due to transient inhibitory feedback mechanisms potentially preventing excessive responses (19). The overall transcriptional upregulation was mirrored at the protein level, with increased expression observed at 24, 48, and 72 hours of co-culture (*P<0.05, **P<0.01, ***P<0.001 vs control groups; **Figure 1B**). The immunosuppressive effects of hDPSCs were confirmed through the analysis of p27 Kip1 and cleaved caspase 3 expression in aPBMCs. As shown in **Figure 2A**, a statistically significant increase of both p27 Kip1 and cleaved caspase 3 was observed in aPBMCs after co-culture with hDPSCs (**P<0.01, ***P<0.001 vs aPBMCs). Introducing a PD-L1-specific inhibitor into the co-culture system resulted in the downregulation of p27 Kip1 and cleaved caspase 3 (^§^P<0.05, ^§§^P<0.01 vs co-cultured aPBMCs; **Figure 2A**), demonstrating that the immunosuppressive effects of hDPSCs are mediated by PD-L1. To increase the translational potential of these observations we assessed the concentrations of 18 cytokines in culture supernatants obtained from aPBMCs with and without co-culture with hDPSCs. For these experiments, PBMCs were isolated from patients affected by RA, who were unresponsive to csDMARDs. Consistently with our recent study (20), hDPSCs cultured alone produced only IL-6 (224 pg/ml ± 75 SEM) among the investigated cytokines. Resting PBMCs produced only IL-6 and IL-10 (data not shown). CD3/CD28 activation of PBMCs resulted in increased levels of all measured cytokines. Notably, co-culture with hDPSCs further elevated the concentrations of IL-6 and GM-CSF, whereas the levels of TNF-α, IL-2, IL-10, IL-12(p70), and IL-23 significantly decreased (*P<0.05 vs aPBMCs cultured alone; **Figures 2B, C**). The concentrations of other cytokines were either unaffected or showed sample-to-sample variability. Altogether, this evidence supports the immunosuppressive effects of hDPSCs mediated by PD-L1 whose potential can be translated to the inflammatory milieu of RA patients.

### PD-L1 correlated to IL-6 expression in hDPSCs after indirect co-culture with aPBMCs

**Figure 3:**
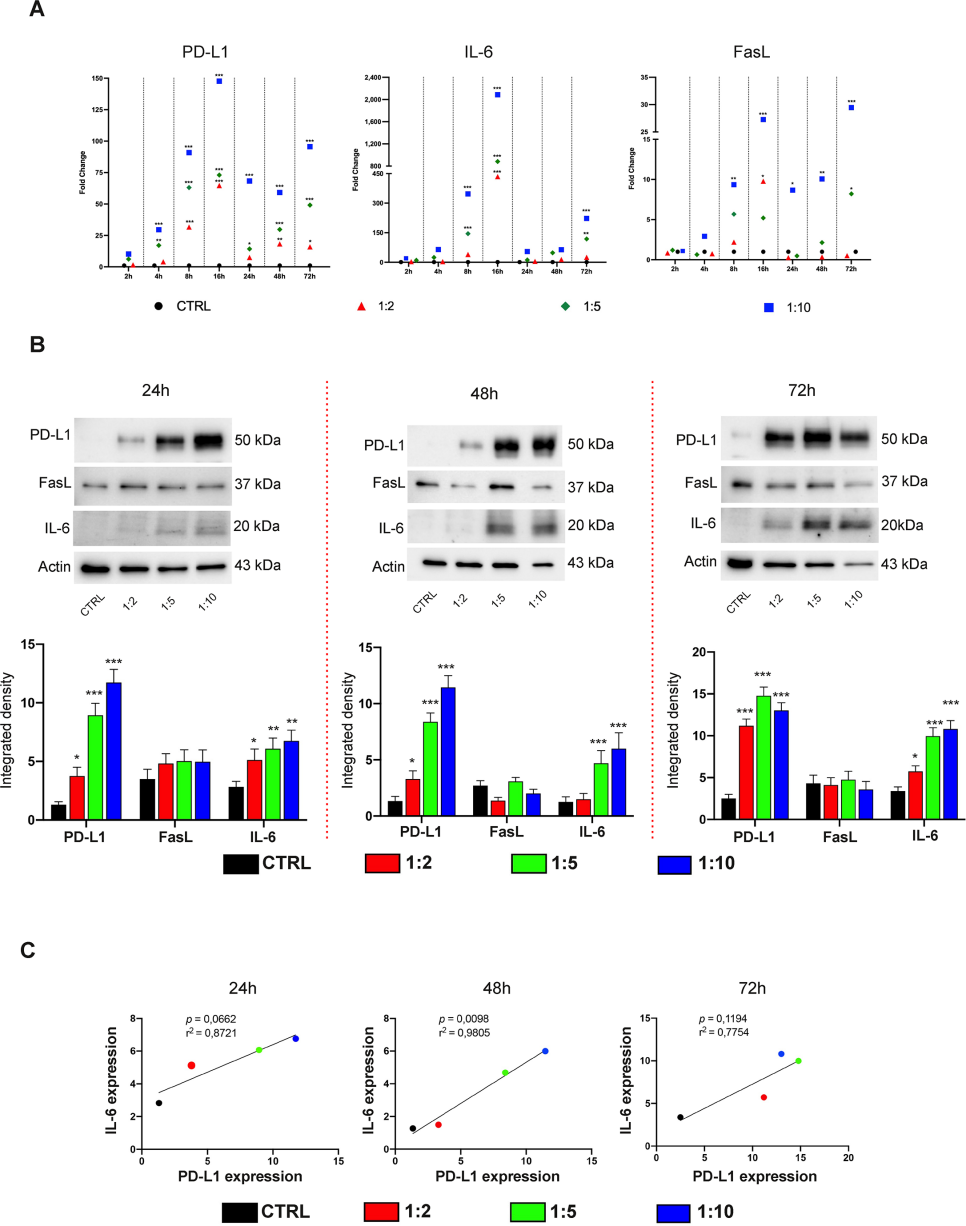
PD-L1 and IL-6 but not FasL are upregulated in hDPSCs following indirect co-culture with aPBMCs. **(A)** PD-L1, IL-6 and FasL mRNA levels were detected in hDPSCs co-cultured with aPBMCs by using a transwell system for 2, 4, 8, 16, 24, 48 and 72 hours. Different shapes are colour-coded to distinguish different hDPSCs-aPBMCs co-culture ratios (1:2, 1:5, 1:10). Data are indicated as mean values of independent experiments using cells from different donors (n=3). **(B)** Analyses of PD-L1, FasL and IL-6 protein expression in hDPSCs after 24, 48 and 72 h of indirect co-culture with aPBMCs (hDPSCs-aPBMCs; 1:2, 1:5, 1:10 ratios). Data are representative of n=3 independent experiments, with mean values ± SD reported in the related densitometric analyses. Original blots are reported in **Supplementary Figure S2C**. In all cases, statistical analyses were carried out by using one-way ANOVA with Tukey’s *post-hoc* test (*P<0.05, **P<0.01, ***P<0.001 vs control group). **(C)** Correlation between PD-L1 and IL-6 protein expression in hDPSCs after 24, 48 and 72 hours of aPBMCs indirect co-culture as determined by western blot densitometric analysis. Dots are colour-coded to indicate 1:2, 1:5, 1:10 hDPSCs-aPBMCs co-culture ratios. P values indicate results of Pearson’s correlation test and R^2^ values the square of the correlation coefficient.

The potential of hDPSCs to modulate the inflammatory milieu via the secretion of soluble factors was also assessed. To this end, the expression of PD-L1, FasL and IL-6 was analyzed in hDPSCs indirectly co-cultured with aPBMCs using a transwell culture system. Our data demonstrated a significant upregulation of PD-L1 and IL-6 expression in hDPSCs following co-culture, showing a time-dependent and co-culture-ratio-dependent modulation (*P<0.05, **P<0.01, ***P<0.001 vs control groups; **Figures 3A, B**; raw data and SD in **Supplementary Table S5**). In particular, PD-L1 and IL-6 mRNA levels reached a peak at 16 hours, followed by a partial down-regulation at 24 hours, and a subsequent increase at later time points (48–72 hours). Under these co-culture conditions, FasL mRNA levels were statistically significantly upregulated only at later time points and mainly at the highest co-culture ratios (*P<0.05, **P<0.01, ***P<0.001 vs control groups; **Figure 3A**, raw data and SD in **Supplementary Table S5**), even though no significant changes in FasL protein expression were observed under these conditions (**Figure 3B**). Overall, these findings suggest that the upregulation of PD-L1 and IL-6 is predominantly mediated by soluble factors, whereas a direct cell-to-cell contact is required to drive FasL expression. Moreover, a significant correlation was observed between PD-L1 and IL-6 expression in hDPSCs after 24 (p=0,0662), 48 (p=0,0098) and 72 (p=0,1194) hours of indirect co-culture (**Figure 3C**).

### IL-6 is involved in regulating PD-L1 expression

**Figure 4:**
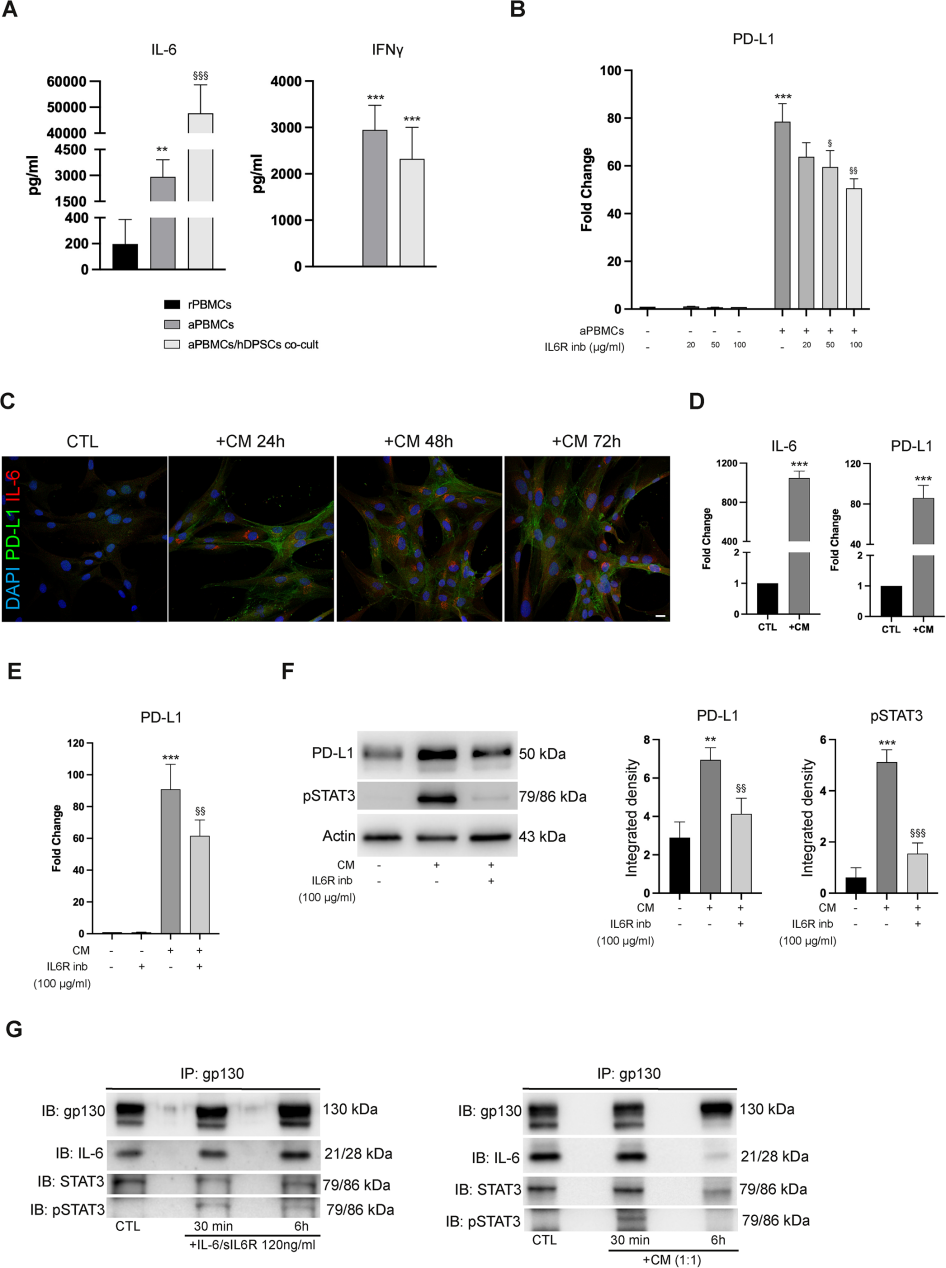
IL-6 is involved in regulating PD-L1 expression. **(A)** IFNγ and IL-6 concentration (pg/ml) detected by multiplex bead-based assay in culture supernatants of rPBMCs, aPBMCs cultured alone and aPBMCs/hDPSCs co-culture for 48 hours. Data are shown as mean values ± SD of independent experiments using cells from different donors (n=3). Statistical analysis was performed by one-way ANOVA followed by Tukey’s *post-hoc* test (**P<0.01, ***P<0.001 vs rPBMCs, ^§§§^P<0.001 vs aPBMCs). **(B)** PD-L1 mRNA levels evaluated in hDPSCs with or without exposure to aPBMCs (1:10 hDPSCs-aPBMCs ratio) and/or IL6R inhibitor (20, 50, 100 µg/ml) for 24 hours. Data are shown as mean ± SD of independent experiments using cells from n=3 donors (***P<0.001 vs hDPSCs alone; ^§^P<0.05, ^§§^P<0.01 vs co-cultured hDPSCs). **(C)** Immunofluorescence images showing PD-L1 (green) and IL-6 (red) expression in hDPSCs treated with or without CM for 24, 48 and 72 hours. Nuclei were counterstained with DAPI. Scale bar: 10 µm. **(D)** Real Time PCR analysis of IL-6 and PD-L1 expression in hDPSCs after exposure to CM for 24h. Results are presented as mean ± SD from n=3 independent experiments (***P<0.001 vs untreated hDPSCs, Student’s t test) **(E)** PD-L1 expression detected in hDPSCs cultured for 24 hours with or without CM and/or IL6R inhibitor (100 µg/ml). Data are shown as mean ± SD of independent experiments using cells from n=3 donors (***P<0.001 vs control group; ^§§^P<0.01 vs CM-treated hDPSCs) **(F)** Western blot analysis of PD-L1 and pSTAT3 expression in hDPSCs with or without exposure to CM and/or IL6R inhibitor (100 µg/ml). Histograms show target densitometric analyses. Error bars represent SD obtained from n=3 independent experiments (**P<0.01, ***P<0.001 vs control group; ^§§^P<0.01, ^§§§^P<0.001 vs CM-treated hDPSCs). Original blots are presented in **Supplementary Figure S2D**. **(G)** Immunoblotting (IB) of gp130, IL-6, STAT3 and pSTAT3 of gp130 immunoprecipitates (IPs) derived from hDPSCs after treatment with either IL-6/sIL6R complex (left) or CM (right) for 30 min and 6h (n=3 independent experiments). Original blots are reported in **Supplementary Figure S3A**. P values of **(A, B, E, F)**: one-way ANOVA with Tukey’s *post-hoc* test.

To further investigate the potential correlation between IL-6 and PD-L1, we firstly investigated IL-6 levels in co-culture supernatants. Interestingly, supernatants from aPBMCs/hDPSCs co-culture displayed increased levels of IL-6 when compared to aPBMCs cultured alone (^§§§^P<0.001 vs aPBMCs; **Figure 4A**), while no significant changes were observed in IFNγ levels when compared to supernatants from aPBMCs (**Figure 4A**). These data suggest that hDPSCs directly influence the surrounding microenvironment by releasing IL-6. Next, a specific inhibitor of IL-6 pathways was included in our experimental setup. hDPSCs were co-cultured with aPBMCs in presence or absence of IL6R inhibitor. Data reported in **Figure 4B** showed that the upregulated PD-L1 mRNA levels induced in hDPSCs after co-culture (***P<0.001 vs hDPSCs alone) were partially reversed in presence of IL6R inhibitor in a dose-dependent manner, reaching statistically significant values at 50 and 100 μg/ml (^§^P<0.05, ^§§^P<0.01 vs CM-treated hDPSCs; **Figure 4B**). To avoid any effects of IL6R inhibitor on aPBMCs due to the IL6R expression on the cell membrane of these mononuclear cells (13), hDPSCs were treated with CM previously collected from aPBMCs. The upregulation of PD-L1 and IL-6 was detected in hDPSCs after 24, 48 and 72 hours of treatment (***P<0.001 vs control group; **Figures 4C, D**). Moreover, the upregulated PD-L1 mRNA levels of CM-treated hDPSCs (***P<0.001 vs untreated hDPSCs; **Figure 4E**) were partially, yet significantly, decreased when IL6R inhibitor was added to the culture system (^§§^P<0.01 vs CM-treated hDPSCs; **Figure 4E**). In addition, western blot analysis showed a downregulation of CM-induced pSTAT3 and PD-L1 levels after IL6R inhibitor treatment (**P<0.01, ***P<0.001 vs untreated hDPSCs; ^§§^P<0.01, ^§§§^P<0.001 vs CM-treated hDPSCs; **Figure 4F**). These data confirm the role of IL-6 in supporting PD-L1 expression in hDPSCs exposed to inflammatory conditions, likely acting in an autocrine manner. To confirm this hypothesis, immunoprecipitation experiments were performed in hDPSCs treated with CM for 30 min and 6 hours (**Figure 4G**). IL-6 trans-signaling pathway was stimulated with IL-6/sIL6R complex and used as a positive control. The activation of IL-6/STAT3 pathway was observed in hDPSCs after 30 minutes of IL-6/sIL6R treatment. In parallel, the same result was detected in CM-treated hDPSCs. Altogether, these data indicate that the IL-6 released by hDPSCs activates the IL-6/STAT3 pathway on hDPSCs themselves, acting in an autocrine manner.

### Activation of IL-6 trans-signalling pathway

**Figure 5:**
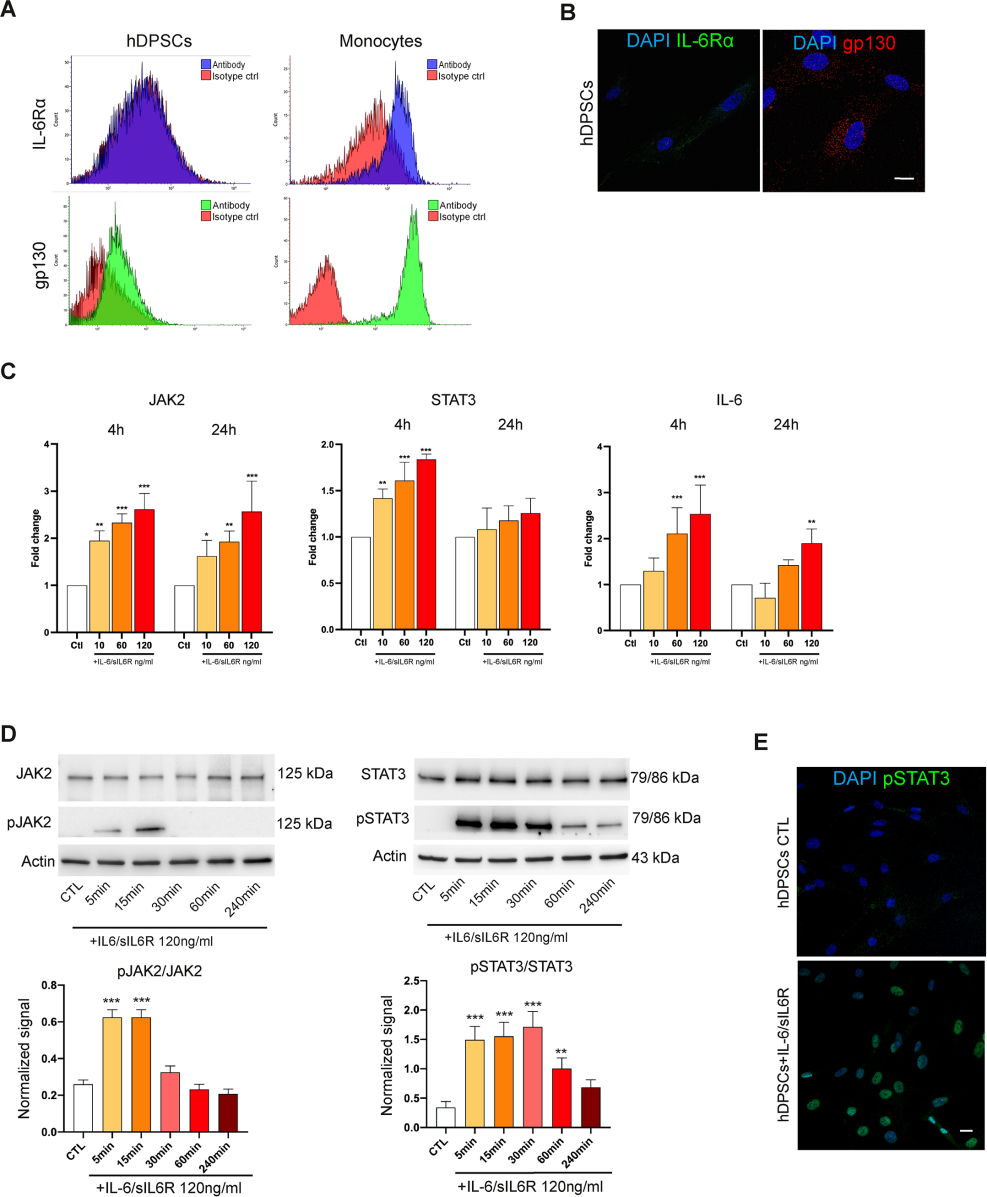
Activation of IL-6 trans-signalling pathway in hDPSCs. **(A)** Cell surface expression of IL6Rα and gp130 in hDPSCs was detected by flow cytometry using monocytes as positive control. **(B)** Immunofluorescence analyses showing IL6Rα (green) and gp130 (red) in hDPSCs. Nuclei were counterstained with DAPI. Scale bar: 10 µm. **(C)** Expression of JAK2, STAT3 and IL-6 detected by Real Time PCR in hDPSCs treated with increasing concentrations of IL-6/sIL6R complex (10, 60, 120 ng/ml) for 4 and 24 hours. Statistical analyses were performed by one-way ANOVA followed by Tukey’s *post-hoc* test within each timepoint (*P<0.05, **P<0.01, ***P<0.001 vs control group). **(D)** Phosphorylation of JAK2 and STAT3 detected by western blot analyses in hDPSCs upon IL-6/sIL6R treatment (120 ng/ml) for 5, 15, 30, 60, 240 minutes. Histograms represent the pJAK2/JAK2 and pSTAT3/STAT3 ratios as determined by densitometric analyses of each target (n=3 independent experiments). Original blots are reported in **Supplementary Figure S3B**. P values indicate statistically significant differences by using a one-way ANOVA with Tukey’s *post-hoc* test (**P<0.01, ***P<0.001 vs control group). **(E)** Nuclear localization of pSTAT3 shown by immunofluorescence analyses carried out on control (CTL) hDPSCs and IL-6/sIL6R-treated hDPSCs (120 ng/ml) for 30 minutes. Nuclei were counterstained with DAPI. Scale bar: 10 µm.

We investigated the activation of the IL-6 pathways in hDPSCs. Flow cytometry analysis showed that hDPSCs expressed only gp130 and not IL-6Rα, as further supported by immunofluorescence analysis (**Figure 5A, B**). hDPSCs were next treated with increasing concentrations of IL-6/sIL6R complex for 4 hours and 24 hours. Histograms reported in **Figure 5C** showed a dose-dependent rising expression of IL-6 upstream (i.e., JAK2 and STAT3) and downstream (IL-6 itself) targets in treated-hDPSCs when compared to the control group (*P<0.05, **P<0.01, ***P<0.001 vs control group; **Figure 5C**). Since these molecules are mainly regulated through posttranslational mechanisms, we investigated phospho JAK2 (pJAK2) and pSTAT3 in hDPSCs treated with IL-6/sIL6R complex (120 ng/ml) at different time points. Western blot analyses showed statistically significant increases of pJAK2/JAK2 and pSTAT3/STAT3 ratios as early as after 5 minutes of stimulation (**P<0.01, ***P<0.001 vs untreated hDPSCs; **Figure 5D**). In addition, pSTAT3 functional nuclear localization was revealed by confocal immunofluorescence analyses (**Figure 5E**). These data demonstrate the activation of the trans-signalling pathway in hDPSCs upon stimulation with IL-6/sIL6R complex.

### IL-6 positively regulates PD-L1 protein stability

**Figure 6:**
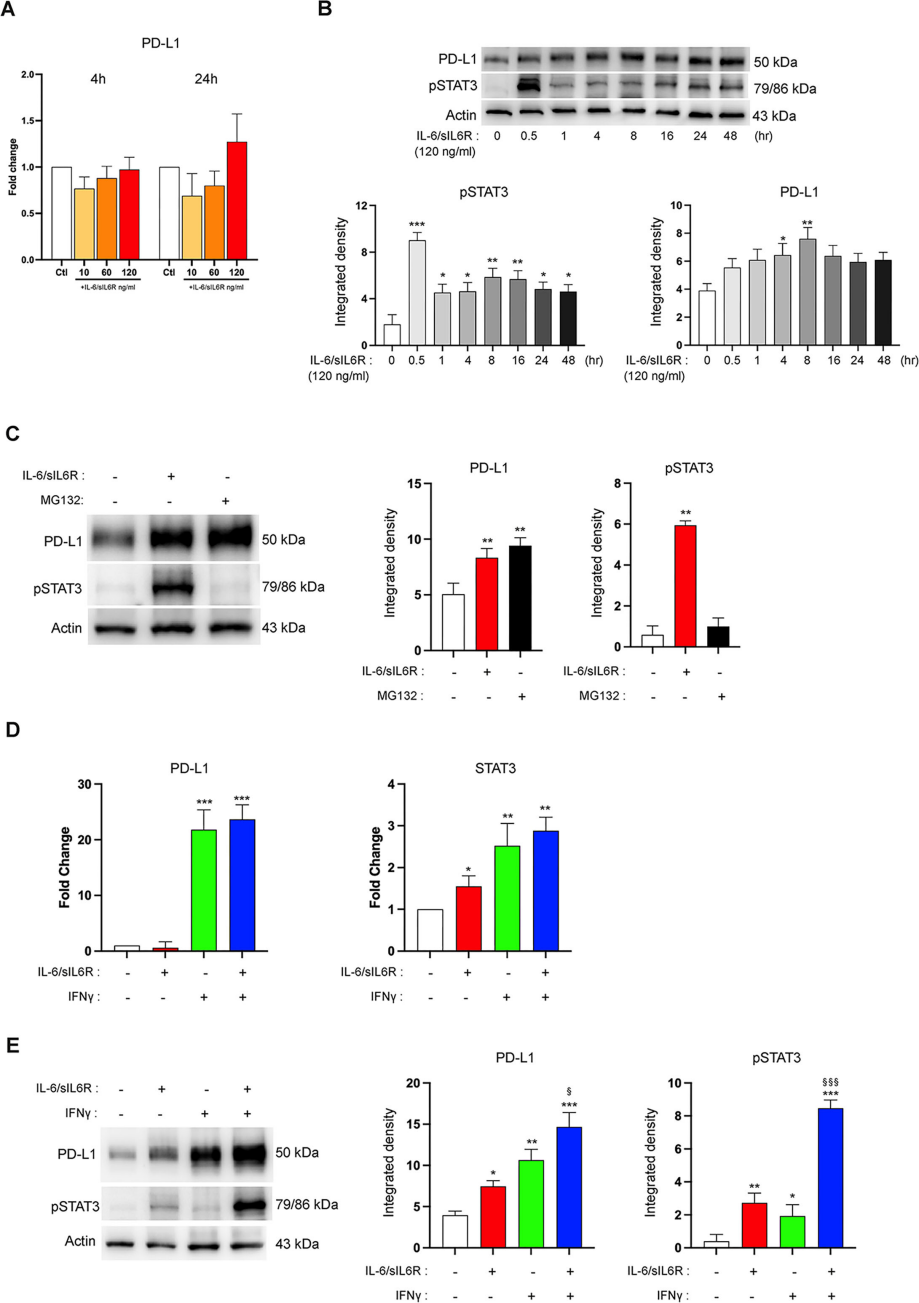
IL-6 positively regulates PD-L1 protein stability. **(A)** PD-L1 mRNA levels investigated in hDPSCs treated with IL-6/sIL6R complex (10, 60, 120 ng/ml) for 4 and 24 hours. **(B)** Western blot analyses showing PD-L1 and pSTAT3 protein expression in hDPSCs following IL-6/sIL6R treatment for 0.5, 1, 4, 8, 16, 24 and 48 hours. Histograms represent the densitometric analysis of PD-L1 and pSTAT3 (*P<0.05, **P<0.01, ***P<0.001 vs control group). Original blots are reported in **Supplementary Figure S3C**. **(C)** PD-L1 and pSTAT3 protein expression in hDPSCs with or without exposure to IL-6/sIL6R complex (120 ng/ml, 8 hours) or MG132 (10 μM, 6 hours). Histograms represent the related densitometric analysis (**P<0.01 vs untreated hDPSCs). Original blots are shown in **Supplementary Figure S3D**. **(D)** Real Time PCR analyses of PD-L1 and STAT3 mRNA expression in IL-6/sIL6R complex (120ng/ml, 8hours) and/or IFNγ (10ng/ml, 8 hours) treated hDPSCs (*P<0.05, **P<0.01, ***P<0.001 vs untreated hDPSCs). **(E)** PD-L1 and pSTAT3 protein expression detected in hDPSCs with or without exposure to IL-6/sIL6R complex (120ng/ml, 8 hours) and/or IFNγ (10ng/ml, 8 hours). Histograms represent the related densitometric analysis (*P<0.05, **P<0.01, ***P<0.001 vs untreated hDPSCs; ^§^P<0.05 vs IFNγ-treated hDPSCs, ^§§§^P<0.001 vs IFNγ-treated hDPSCs). Original blots are presented in **Supplementary Figure S3E**. Results are presented as mean ± SD of n=3 independent experiments. P values indicate statistically significant differences from one-way ANOVA with Tukey’s *post-hoc* test.

Since the IL-6 trans-signaling pathway can be activated in hDPSCs and a positive correlation between IL-6 and PD-L1 occurred in hDPSCs exposed to an inflammatory microenvironment, we investigated the mechanism of IL-6 in modulating PD-L1 expression. As reported in **Figure 6A**, PD-L1 mRNA levels did not show any significant modulation in hDPSCs treated with IL-6/sIL6R complex regardless of doses and time of exposure. Meanwhile, treatment of hDPSCs with IL-6/sIL6R complex induce the expression of pSTAT3 and a significant increase of PD-L1 protein expression after 4 and 8 hours (*P<0.05, **P<0.01, ***P<0.001 vs untreated hDPSCs; **Figure 6B**). These data suggest that the IL-6 trans-signaling positively regulated PD-L1 protein stability via a non-transcriptional regulatory mechanism such as posttranslational regulation. Treatment with MG132, a proteasome inhibitor, significantly upregulated PD-L1 protein level (**P<0.01 vs untreated hDPSCs; **Figure 6C**, lane 3). Interestingly, IL-6/sIL6R treatment induced PD-L1 expression to a similar degree to that observed in hDPSCs treated with MG132, even though pSTAT3 expression was detected only in IL-6/sIL6R treated cells (**P<0.01 vs untreated hDPSCs; **Figure 6C**, lane 2). The role of IL-6 to affect PD-L1 expression via posttranslational mechanism was further validated by comparing the IL-6/sIL6R effect to IFNγ, known to be the most potent inducer of PD-L1 (21). Unlike the induction of PD-L1 mediated by IFNγ via transcription activation (**P<0.01, ***P<0.001 vs untreated hDPSCs; **Figure 6D, E**, lane 3), the action of IL-6/sIL6R complex on PD-L1 expression was primarily at the posttranscriptional level (*P<0.05 vs untreated hDPSCs; **Figure 6E**, lane 2) as IL-6/sIL6R treatment did not influence PD-L1 mRNA expression (**Figure 6D**). In addition, IL-6/sIL6R and IFNγ co-treatment empowers PD-L1 protein expression in hDPSCs (^§^P<0,05 vs IFNγ-treated hDPSCs; **Figure 6E**, lane 4), while mRNA level did not change (**Figure 6D**). In parallel, pSTAT3 levels further increased when hDPSCs were co-stimulated with IL-6/sIL6R and IFNγ (^###^P<0.001 vs IL-6/sIL6R-treated hDPSCs; **Figure 6E**) suggesting a cooperative effect (22). Overall, these data support the differential mechanism by which IL-6 and IFNγ enhance PD-L1 expression in hDPSCs.

### IL-6 is not crucial for PD-L2 upregulation in hDPSCs

**Figure 7:**
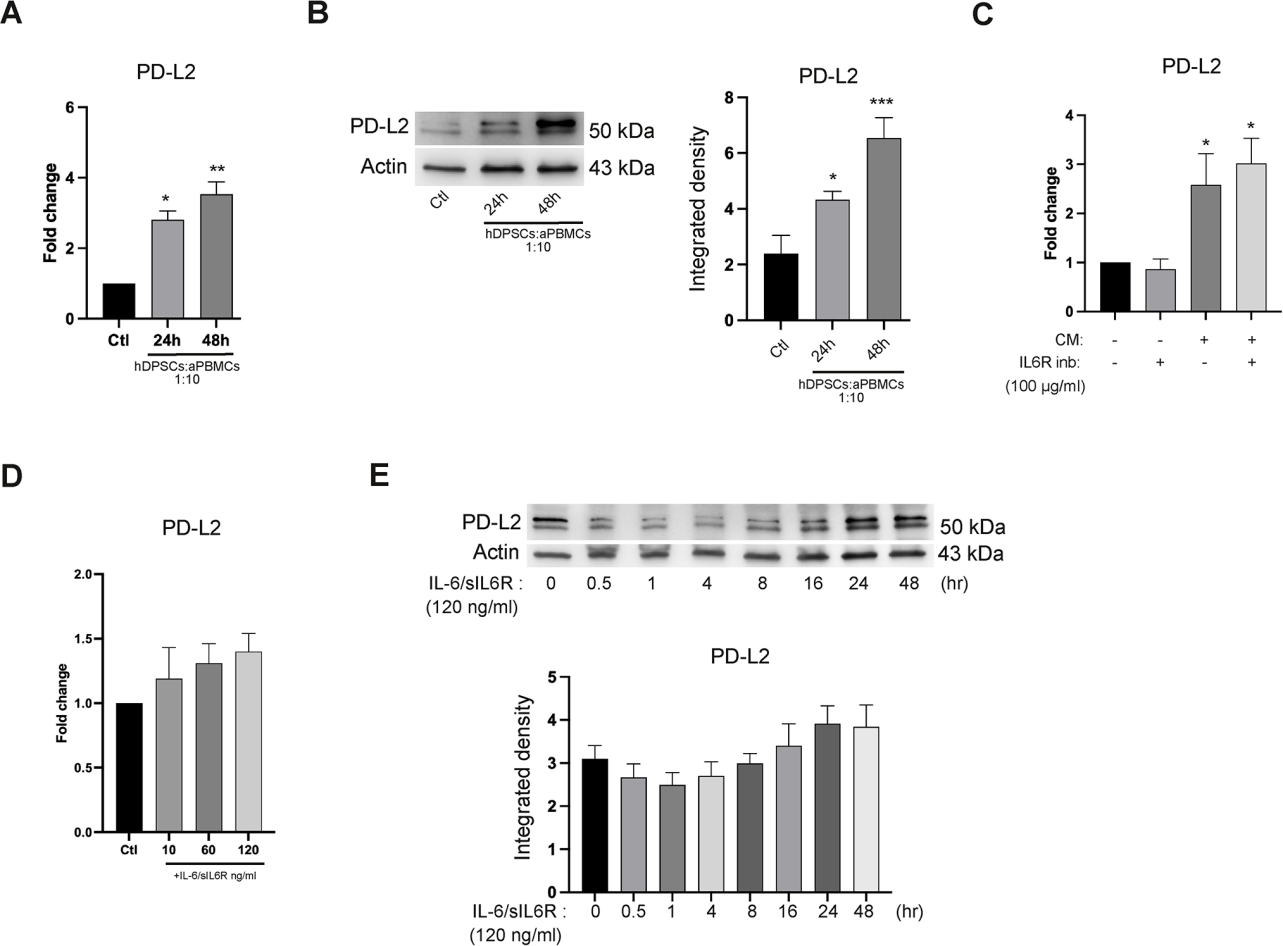
Effects of IL-6 on PD-L2 expression. **(A)** PD-L2 mRNA levels investigated in hDPSCs indirectly co-cultured with aPBMCs (hDPSCs-aPBMCs, 1:10 ratio) for 24 and 48 hours. **(B)** Western blot analyses showing PD-L2 protein expression in hDPSCs following co-culture with aPBMCs (hDPSCs-aPBMCs, 1:10 ratio) for 24 and 48 hours. Histograms represent its densitometric analysis. Original blots are reported in **Supplementary Figure S4A**. **(C)** Real Time PCR analysis of PD-L2 expression in hDPSCs cultured for 24 hours with or without CM and/or IL6R inhibitor (100 µg/ml). Data of 7A-B-C are shown as mean ± SD of independent experiments using cells from n=3 donors (*P<0.05, **P<0.01, ***P<0.001 vs control group). **(D)** PD-L2 mRNA expression evaluated in IL-6/sIL6R complex (10, 60, 120 ng/ml, 4 hours) treated hDPSCs. **(E)** PD-L2 protein expression detected in hDPSCs exposed to IL-6/sIL6R complex (120 ng/ml) for 0.5, 1, 4, 8, 16, 24, 48 hours. Histograms represent the related densitometric analysis. Original blots are presented in **Supplementary Figure S4B**. Results of **(D, E)** are presented as mean ± SD of n=3 independent experiments. In any case, P values indicate statistically significant differences from one-way ANOVA with Tukey’s *post-hoc* test.

To broaden our knowledge on the regulatory mechanisms associated with the activation of IL-6/JAK/STAT3 pathway in hDPSCs, we first evaluated the expression of PD-L2 in hDPSCs under inflammatory conditions. Following indirect co-culture with aPBMCs, hDPSCs increased the expression of PD-L2 (*P<0.05, **P<0.01, ***P<0.001 vs control group; **Figures 7A, B**), revealing that hDPSCs take advantage of PD-L2 to mediate their immunomodulatory effects. Based on these findings, we examined the involvement of IL-6 in the upregulation of PD-L2. Inhibition of the IL-6 pathway did not alter the increased PD-L2 levels induced by CM treatment of hDPSCs (*P<0.05 vs control group; **Figure 7C**). Similarly, stimulation of the IL-6 pathway did not influence PD-L2 mRNA levels across all concentrations of the IL-6/sIL6R complex tested (**Figure 7D**). Moreover, treatment of hDPSCs with the highest concentration of IL-6/sIL6R complex did not result in any significant change in PD-L2 protein expression (**Figure 7E**), suggesting that IL-6/JAK/STAT3 signaling selectively enhances PD-L1 expression without affecting PD-L2.

## Discussion

The intricate interplay between the MSC immunomodulatory properties and the inflammatory process has been well established. The immunoregulatory function of MSCs is highly context-dependent, influenced by the dynamic microenvironment, which fluctuates throughout the immune response due to factors such as the duration, type, and concentration of inflammatory mediators, as well as the involvement of distinct immune cell subtypes (1, 23–25). Our previous studies demonstrated that hDPSCs exposed to inflammatory conditions can modulate immune responses by regulating the release of pro-inflammatory cytokines and by exploiting both Fas/FasL and PD1/PD-L1 pathways in a synergistic manner (2, 7, 12). Additionally, extracellular vesicles (EVs) have gained increasing attention as paracrine signaling entities able to modulate T-cell responses and cell differentiation (26, 27). In the present study, we first demonstrate that soluble mediators, mimicking the acute phase of inflammatory diseases (28), act as a trigger for the upregulation of both PD-L1 and PD-L2 in hDPSCs indirectly co-cultured with aPBMCs. Our data also demonstrate that cell-cell contact further potentiates the activation of PD1/PD-L1 and Fas/FasL pathways in hDPSCs, reinforcing their synergistic role, consistent with our prior findings (2, 7). Moreover, we have shown in our previous work that the exposure to aPBMCs derived from RA patients induces PD-L1 expression in hDPSCs, supporting the relevance of this pathway in their immunomodulatory function (2). In line with these findings, here we provided evidence that co-culturing hDPSCs with RA aPBMCs leads to a reduction in the secretion of inflammatory cytokines, indicating that hDPSCs can modulate a pathological inflammatory microenvironment. However, hDPSCs were unable to regulate IL-6 secretion, with its levels increasing following co-culture. A similar pattern has also been observed in hDPSCs co-cultured with aPBMCs derived from COVID-19 patients (6). This discrepancy is significant and requires further clarification, as IL-6 plays a central role in inflammation and immune regulation. Recent evidence has highlighted that IL-6 can contribute to the immunosuppressive functions of MSCs (14–18). In fact, IL-6 knockdown has been shown to impair the immunosuppressive capacity of BM-MSCs (15), while IL-6 overexpression appears to enhance these properties (18). Here we demonstrate that under inflammatory conditions, hDPSCs upregulate IL-6 expression, suggesting that IL-6 may be involved in modulating their immunomodulatory behavior. Elucidating whether and how IL-6 influences hDPSCs’ immunoregulatory properties could provide valuable insights into their potential applications in inflammatory diseases. A key novel finding from our study is the identification of a positive correlation between PD-L1 expression and IL-6 upregulation in hDPSCs upon exposure to an inflammatory microenvironment, a relationship that has not been previously described in these cells. We demonstrated that hDPSCs undergo IL-6 trans-signaling activation via sIL6R, which is typically secreted by activated immune cells (13, 29). Treatment of hDPSCs with the IL-6/sIL6R complex led to activation of the IL-6/JAK2/STAT3 pathway, subsequently upregulating IL-6 itself. Notably, immunoprecipitation experiments revealed that IL-6 produced and secreted by hDPSCs in inflammatory conditions exerts an autocrine effect, reinforcing the IL-6/JAK/STAT3 signaling loop. These findings suggest that the inflammatory microenvironment serves as a key driver of IL-6/JAK/STAT3 activation in hDPSCs, which, in turn, establishes a positive feedback loop that sustains IL-6 expression and potentially influences PD-L1 regulation. Functional inhibition of IL-6 signaling within the inflammatory milieu affects PD-L1 expression, suggesting that IL-6 contributes to the regulation of PD-L1 in hDPSCs, likely together with other soluble factors present in the inflammatory microenvironment.

The ability of IL-6 to influence PD-L1 and PD-L2 expression has been mainly studied in other cellular contexts (30–34). For instance, in hepatocellular carcinoma cells, IL-6 was shown to activate the JAK1 pathway, leading to phosphorylation of PD-L1 whose glycosylation ultimately enhances its stability (30). Similarly, in monocytes and macrophages, IL-6 upregulates PD-L1 expression through the STAT3/c-MYC/miR-25-3p pathway (32). IL-6 has also been reported to regulate PD-L2 expression in head and neck squamous cell carcinoma (34). Our results unveiled a novel regulatory mechanism to hDPSCs in which IL-6/sIL6R treatment enhances PD-L1 protein stability without affecting its mRNA levels. This suggests that posttranslational mechanisms contribute to PD-L1 regulation in hDPSCs. Given that the ubiquitin-proteasome system plays a fundamental role in modulating PD-L1 protein turnover, its disruption could lead to sustained PD-L1 expression (35). Consistently, we observed that proteasome inhibition in hDPSCs resulted in elevated PD-L1 protein levels, mirroring the effects of IL-6/sIL6R treatment. Furthermore, co-stimulation with IL-6/sIL6R and IFNγ led to an even greater increase in PD-L1 protein levels, supporting the notion that both transcriptional and posttranslational mechanisms are activated in hDPSCs under inflammatory conditions. This cooperative effect of IFNγ and IL-6 in enhancing PD-L1 expression aligns with recent studies reinforcing the immunosuppressive function of IL-6 in MSCs (15, 18).This IL-6-driven protein stabilization mechanism specifically enhances PD-L1 expression, without affecting PD-L2. In fact, no changes of PD-L2 expression were observed in hDPSCs when the IL-6 pathway was either stimulated or inhibited. This specificity contrasts with other MSCs, where IL-6 signaling has been reported to influence both PD-L1 and PD-L2 expression, even though no evidence on the underlying mechanism has been provided (14). The mechanistic link between IL-6 and PD-L1 stability observed in hDPSCs might represent, therefore, a defining characteristic of these cells, suggesting a potential peculiarity in the immune regulatory properties of hDPSCs compared to other MSC populations.

## Conclusions

In summary, our findings elucidate a novel role of IL-6 in stabilizing PD-L1 expression in hDPSCs within an inflammatory microenvironment. We propose that IL-6 not only acts as a soluble mediator regulating PD-L1 expression but also sustains its own signaling via an autocrine loop, reinforcing the PD-L1-mediated immunomodulatory potential of hDPSCs. In addition to the upregulation of PD-L1, we demonstrate that hDPSCs exposed to inflammation also upregulate PD-L2. However, the mechanism by which IL-6 promotes the stability of PD-L1 is unique to this molecule and does not extend to PD-L2. These insights pave the way for further investigation into the IL-6/STAT3/PD-L1 axis in various pathological conditions, where its dysregulation may contribute to immune evasion and disruption of tissue homeostasis.

## Data Availability

The original contributions presented in the study are included in the article/**Supplementary Material**. Further inquiries can be directed to the corresponding author/s.
