# Supplementary material for: Immunomodulatory properties of human dental pulp stromal cells: the role of IL-6/JAK/STAT3 pathway and PD-L1
Source: Front Immunol. 2026 Jan 30;17:1713704. doi: 10.3389/fimmu.2026.1713704 (PMC12900704; doi:10.3389/fimmu.2026.1713704)
Supplement: Supplementary file 1 [file DataSheet1.pdf]

Immunomodulatory properties of human dental pulp stromal cells: the role of IL-6/JAK/STAT3 pathway and PD-L1

Rosanna Di Tinco[1], Alessandra Pisciotta[1], Giulia Bertani[1], Giulia Orlandi[1], Laura Bertoni[1], Elisa Pignatti[1], Martina Bonacini[2], Alessandro Rossi[2], Anke J. Roelofs[3], Stefania Croci[2], Cosimo De Bari[3], Carlo Salvarani[1,4*], Gianluca Carnevale[1*]

[1]*Department of Surgery, Medicine Dentistry and Morphological Sciences with Interest in Transplant, Oncology, and Regenerative Medicine, University of Modena and Reggio Emilia, Modena, Italy.*

[2]*Clinical Immunology, Allergy and Advanced Biotechnologies Unit, Azienda Unità Sanitaria Locale-IRCCS di Reggio Emilia, Reggio Emilia, Italy.*

[3]*Rheumatology Research Group, Institute of Genetics and Cancer, University of Edinburgh, Edinburgh, UK*

[4]*Rheumatology Unit, Azienda Unità Sanitaria Locale-IRCCS di Reggio Emilia, Reggio Emilia, Italy.*

***Corresponding authors: carlo.salvarani@unimore.it; gianluca.carnevale@unimore.it**

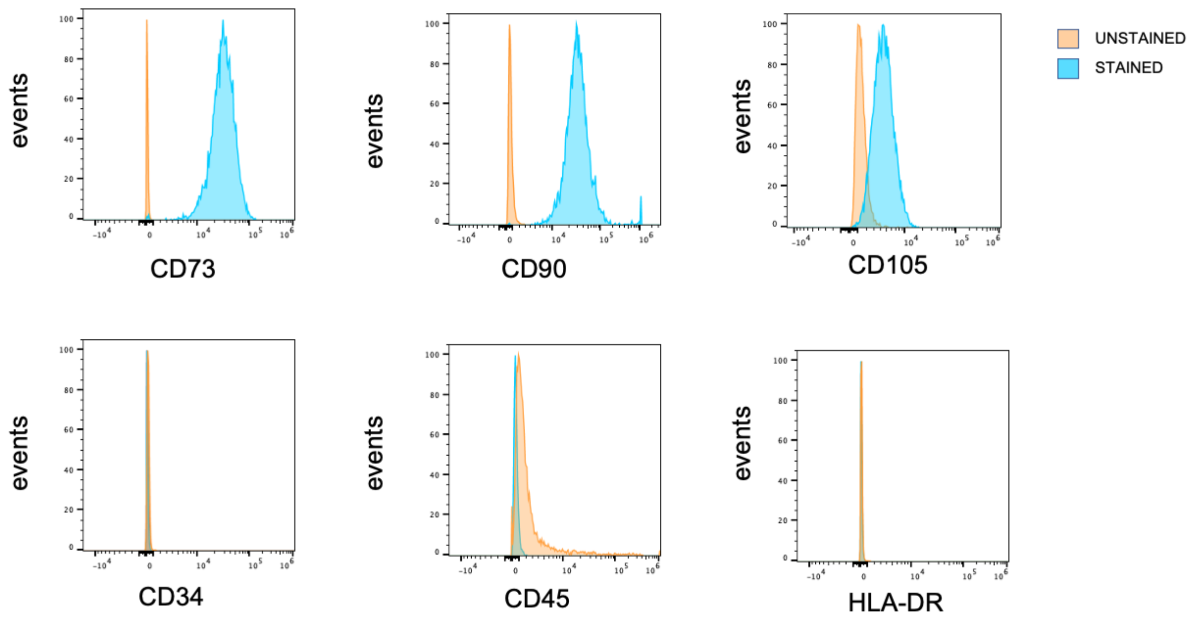

Supplementary figure S1. MSC markers expression in immune-selected hDPSCs. FACS analysis showed that the STRO-1+/c-kit+ hDPSCs express the MSCs typical markers (CD73, CD90, CD105), while lacking the expression of CD34, CD45, HLA-DR. Representative of cells from n=3 subjects.

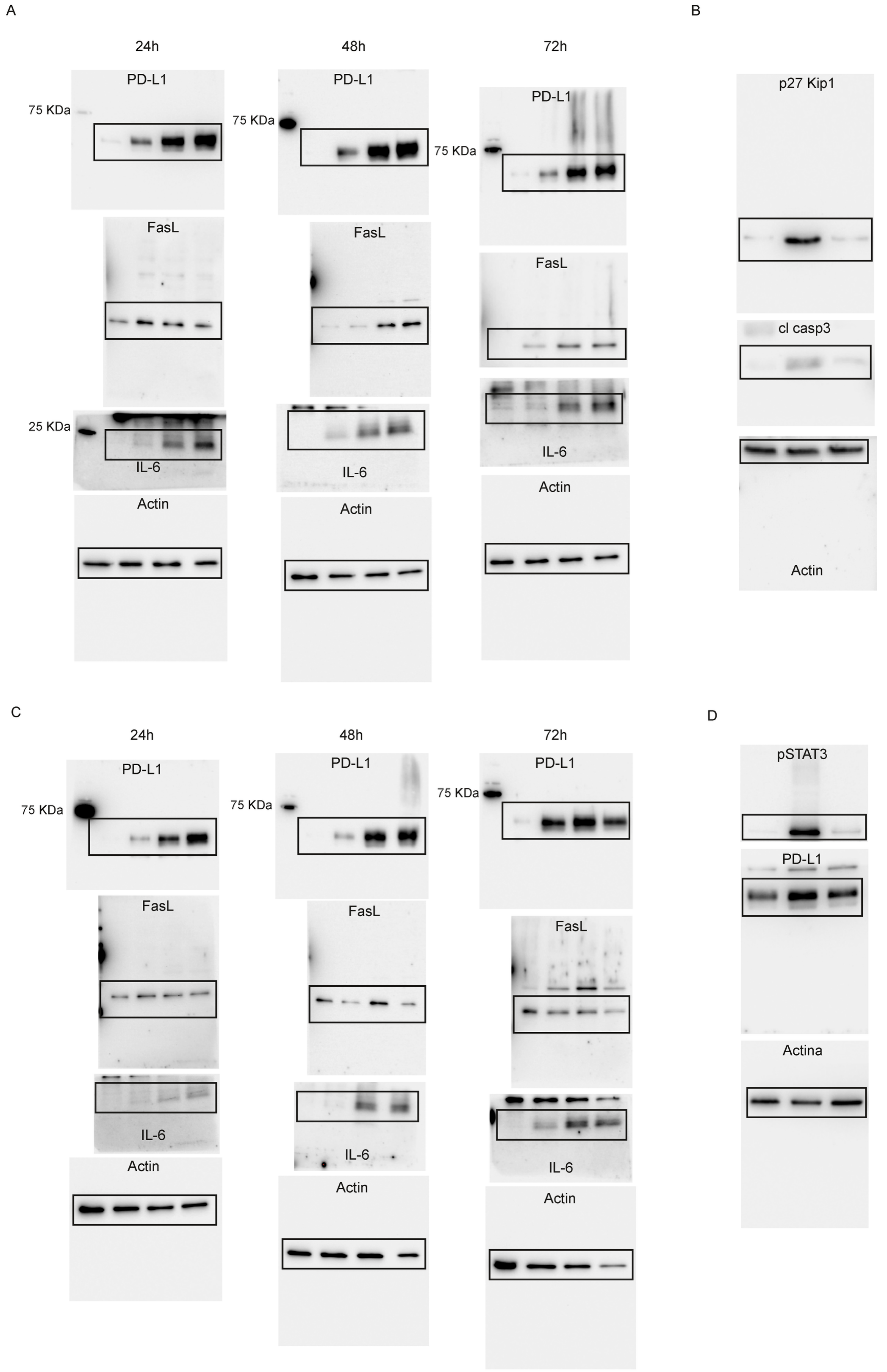

Supplementary figure S2. Extended data for figure 1B, 2A, 3B and 4F. A) Uncropped Western blot images showing PD-L1, FasL, IL-6 and related actin in hDPSCs after direct co-culture with aPBMCs; B) Uncropped Western blot images reporting p27 Kip1, cleaved caspase 3 and related actin in aPBMCs after direct co-culture with hDPSCs; C) Uncropped Western blot images of PD-L1, FasL, IL-6 and related actin in hDPSCs after indirect co-culture with aPBMCs; D) STAT3 phosphorilation (pSTAT3), PD-L1 expression and related actin. Boxed areas correspond to cropped regions shown in figure 1B, 2A, 3B and 4F. Specific target bands were selected according to the molecular weight reported in antibodies' datasheets.

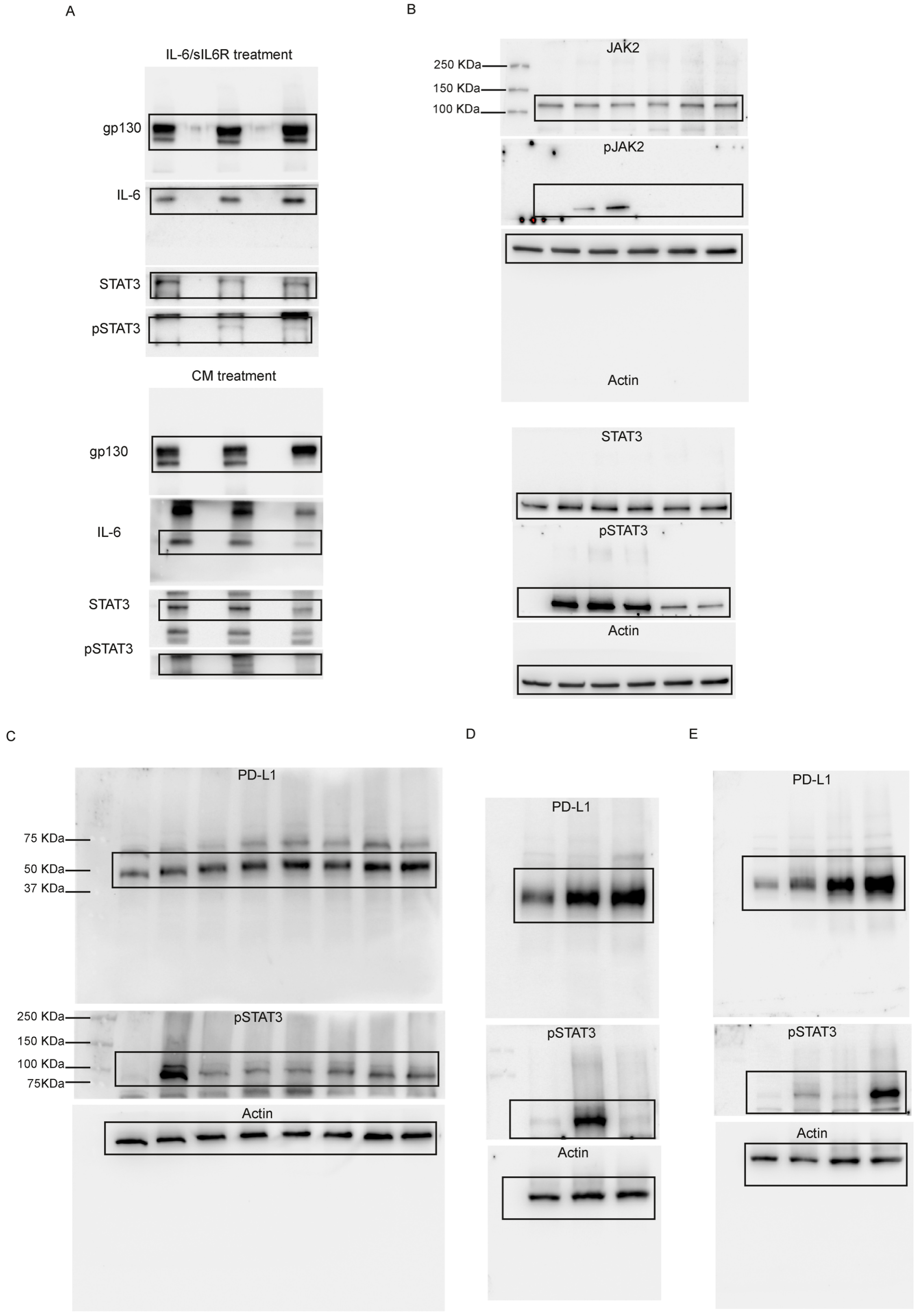

Supplementary figure S3. Extended data for figure 4G, 5D and 6B,C,E. A) Immunoblotting of gp130, IL-6, STAT3 and pSTAT3 of gp130 immunoprecipitates; B) Uncropped Western blot images showing total and phosphorylated JAK2 and STAT3 and the related actin; C-D-E) PD-L1, pSTAT3 and related actin. Boxed areas correspond to cropped regions shown in figure 4G, 5D and 6B,C,E. Specific target bands were selected according to the molecular weight reported in antibodies' datasheets.

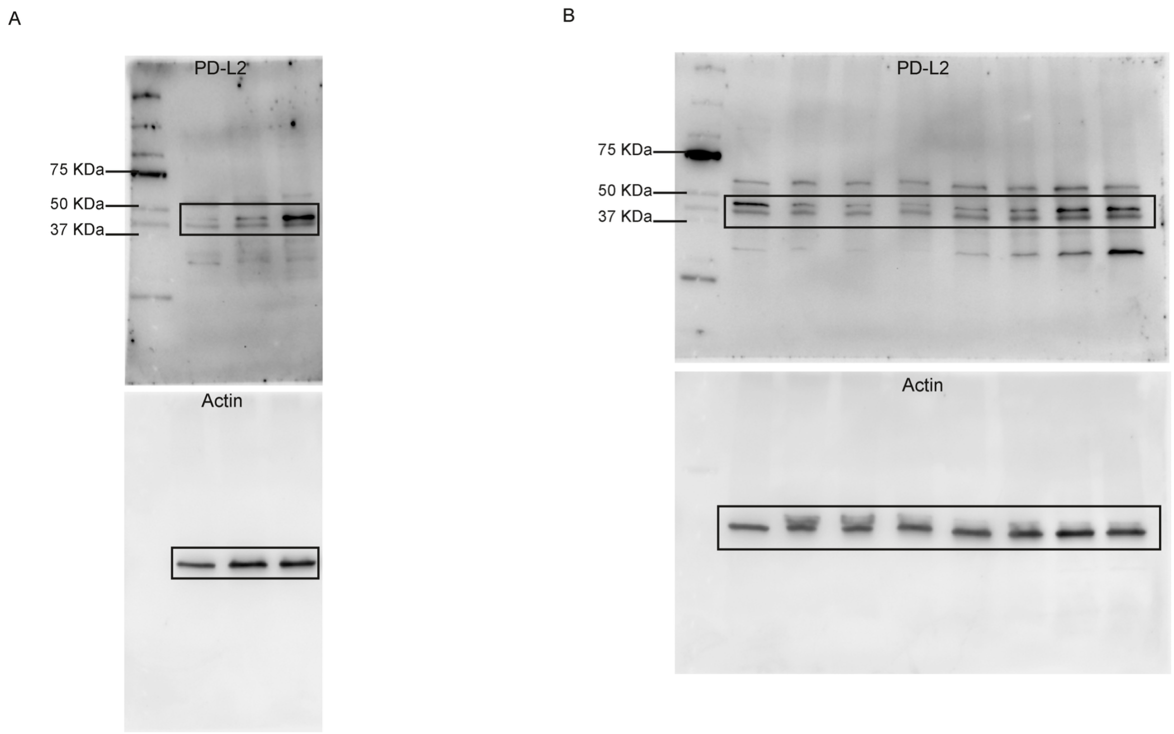

Supplementary figure S4. Extended data for figure 7B,E. A-B) Uncropped Western blot images showing PD-L2 expression and the related actin. Boxed areas correspond to cropped regions shown in figure 7B,E. Specific target bands were selected according to the molecular weight reported in antibodies' datasheets.

Supplementary Table 1. Antibodies for flow cytometry.

Antibody	Clone	Manufacturer	Cat. No.	Conjugation
IL-6Rα	47.7G7.1F2	eBioscience	46-1269-42	PerCP-eFluor™ 710
gp130	2E1B02	BioLegend	362003	PE

Supplementary Table 2. qPCR primer sequences.

Gene	Sequence 5'-3'	
	Forward primer	Reverse primer
Human PD-L1	GGTTGTGGATCCAGTCACCT	TTGGTGGTGGTGGTCTTACC
Human PD-L2	AGGGAAGTGAACAGTGCTATC	GCCAGGTGTTGGCTAGTCTT
Human FasL	AAAGGAGCTGAGGAAAGTGG	CATAGGTGTCTTCCCATTCCAG
Human IL6	AGCCACTCACCTCTTCAGAACGAA	AGTGCCTCTTTGCTGCTTTCACAC
Human JAK2	CCAGATGGAAACTGTTCGCTCAG	GAGGTTGGTACATCAGAAACACC
Human STAT3	CTTTGAGACCGAGGTGTATCACC	GGTCAGCATGTTGTACCACAGG
Human RPLP0	TACACCTTCCCACTTGCTGA	CCATATCCTCGTCCGACTCC

Supplementary Table 3. Antibodies for western blotting and immunoprecipitation.

Antibody	Clone	Manufacturer	Cat. No.	Conjugation
PD-L1	polyclonal	Novus Biologicals	NBP-15791	unconjugated
PD-L2	PDL2/2676	Antibodies.com	A253570	unconjugated
FasL	polyclonal	Santa Cruz	Sc-6237	unconjugated
IL-6	D3K2N	Cell Signaling	12153	unconjugated
pJAK2 (Tyr1007/1008)	C80C3	Cell Signaling	3776	unconjugated
JAK2	D2E12	Cell Signaling	3230	unconjugated
pStat3	D3A7	Cell Signaling	9145	unconjugated
Stat3	124H6	Cell Signaling	9139	unconjugated
gp130	EPR24557-50	Abcam	ab283685	unconjugated
Cleaved Caspase-3	5A1E	Cell Signaling	9664	unconjugated
p27 Kip 1	Y236	Abcam	ab32034	unconjugated
Rabbit IgG isotype control	DA1E	Cell Signaling	3900	unconjugated
β-Actin	C4	Santa Cruz	Sc-47778	unconjugated

Supplementary Table 4. Antibodies for immunofluorescence staining

Antibody	Clone	Manufacturer	Cat. No.	Conjugation
PD-L1	polyclonal	Novus Biologicals	NBP-15791	unconjugated
IL-6	polyclonal	Sigma Aldrich	SAB1400139	unconjugated
IL-6Rα	polyclonal	Abcam	ab128008	unconjugated
gp130	EPR24557-50	Abcam	ab283685	unconjugated
pSTAT3 (Tyr705)	D3A7	Cell Signaling	9145	unconjugated
Rabbit IgG isotype control	polyclonal	R&D Systems	AB-105-C	unconjugated
Donkey anti-mouse IgG	polyclonal	Life Technology	A10036	Alexa Fluor 546
Goat anti-rabbit IgG	polyclonal	Life Technology	A-11070	Alexa Fluor 488

Supplementary Table 5. Extended data for figure 1A and 3A

PD-L1 – Direct contact			
		**Mean**	**SD**
2h	CTRL	1	±0
	1:2	2,29	±0,54
	1:5	3,09	±1,12
	1:10	9,01	±2,57
4h	CTRL	1	±0
	1:2	4,21	±1,75
	1:5	17,17	±3,55
	1:10	34,6	±7,29
8h	CTRL	1	±0
	1:2	23,25	±1,99
	1:5	95,18	±15,62
	1:10	116,73	±19,1
16h	CTRL	1	±0
	1:2	37,99	±6,74
	1:5	79,75	±14,06
	1:10	107,2	±15,88
24h	CTRL	1,00	±0
	1:2	23,46	±4,83
	1:5	46,19	±6,10
	1:10	61,09	±8,28
48h	CTRL	1,00	0,00
	1:2	30,70	±1,83
	1:5	69,91	±7,73
	1:10	84,50	±10,74
72h	CTRL	1,00	±0
	1:2	47,61	±6,14
	1:5	82,01	±8,59
	1:10	101,55	±13,54

IL-6 – Direct contact			
		**Mean**	**SD**
2h	CTRL	1	±0
	1:2	2,47	±0,39
	1:5	5,27	±1,45
	1:10	15,79	±3,84
4h	CTRL	1	±0
	1:2	4,39	±1,05
	1:5	30,93	±5,51
	1:10	97,13	±10,17
8h	CTRL	1	±0
	1:2	8,43	±2,34
	1:5	207,44	±16,1
	1:10	561,92	±35,04
16h	CTRL	1	±0
	1:2	101,43	±4,33
	1:5	826,82	±58,35
	1:10	1415,67	±52,79
24h	CTRL	1	±0
	1:2	36,61	±6,41
	1:5	144,04	±8,11
	1:10	255,51	±5,59
48h	CTRL	1	±0
	1:2	89,81	±10,07
	1:5	310,41	±15,03
	1:10	520,18	±23,6
72h	CTRL	1	±0
	1:2	148,54	±9,86
	1:5	402,52	±70,34
	1:10	763,11	±30,71

FasL – Direct contact			
		**Mean**	**SD**
2h	CTRL	1	±0
	1:2	9,18	±2,24
	1:5	32,3	±3,11
	1:10	37,41	±1,82
4h	CTRL	1	±0
	1:2	19,21	±6,42
	1:5	72,48	±10,53
	1:10	140,36	±5,06
8h	CTRL	1	±0
	1:2	111,81	±18,85
	1:5	834,06	±16,31
	1:10	2034,56	±731,13
16h	CTRL	1	±0
	1:2	383,62	±29,16
	1:5	1551,88	±161,47
	1:10	3894,65	±225,64
24h	CTRL	1	±0
	1:2	37,1	±3,26
	1:5	173,64	±13,92
	1:10	453,39	±66,21
48h	CTRL	1	±0
	1:2	121,09	±7,26
	1:5	729,05	±20,87
	1:10	1311,29	±94,86
72h	CTRL	1	±0
	1:2	470,2	±59,32
	1:5	2138,57	±19,75
	1:10	3958,64	±138,33

PD-L1 – Indirect contact			
		**Mean**	**SD**
2h	CTRL	1	±0
	1:2	1,54	±0,46
	1:5	6,19	±1,07
	1:10	10,21	±2,12
4h	CTRL	1	±0
	1:2	4,05	±1,14
	1:5	17,06	±3,3
	1:10	29,56	±5,65
8h	CTRL	1	±0
	1:2	31,66	±6,75
	1:5	63,08	±8,54
	1:10	90,84	±16,57
16h	CTRL	1	±0
	1:2	64,7	±5,81
	1:5	73,05	±6,24
	1:10	147,68	±13,56
24h	CTRL	1	±0
	1:2	7,48	±2,17
	1:5	14,26	±3,4
	1:10	68,26	±7,36
48h	CTRL	1	±0
	1:2	18,24	±6,59
	1:5	29,75	±9,82
	1:10	59,16	±8,16
72h	CTRL	1	±0
	1:2	15,92	±2,41
	1:5	49,12	±7,81
	1:10	95,59	±11,39

IL-6 – Indirect contact			
		**Mean**	**SD**
2h	CTRL	1	±0
	1:2	3,18	±0,71
	1:5	10,13	±2,18
	1:10	19,96	±5,38
4h	CTRL	1	±0
	1:2	2,99	±0,44
	1:5	24,37	±4,02
	1:10	64,66	±5,06
8h	CTRL	1	±0
	1:2	39,55	±8,93
	1:5	145,09	±15,64
	1:10	346,7	±13,14
16h	CTRL	1	±0
	1:2	434,8	±12,06
	1:5	881,39	±56,15
	1:10	2086,75	±102,17
24h	CTRL	1	±0
	1:2	4,66	±0,92
	1:5	11,72	±3,03
	1:10	54,22	±3,39
48h	CTRL	1	±0
	1:2	14,22	±2,55
	1:5	48,18	±2,57
	1:10	63,77	±30,02
72h	CTRL	1	±0
	1:2	25,26	±5,53
	1:5	118,38	±44,84
	1:10	222,26	±22,17

FasL – Indirect contact			
		**Mean**	**SD**
2h	CTRL	1	±0
	1:2	0,84	±0,11
	1:5	1,19	±0,43
	1:10	1,09	±0,88
4h	CTRL	1	±0
	1:2	0,75	±0,71
	1:5	0,64	±0,34
	1:10	2,92	±3,06
8h	CTRL	1	±0
	1:2	2,19	±0,87
	1:5	5,68	±0,44
	1:10	9,35	±0,97
16h	CTRL	1	±0
	1:2	9,78	±6,81
	1:5	5,2	±2,12
	1:10	27,26	±7,74
24h	CTRL	1	±0
	1:2	0,27	±0,14
	1:5	0,48	±0,33
	1:10	8,67	±2,23
48h	CTRL	1	±0
	1:2	0,34	±0,21
	1:5	2,14	±0,37
	1:10	10,06	±1,79
72h	CTRL	1	±0
	1:2	0,51	±0,16
	1:5	8,21	±1,65
	1:10	29,45	±5,45
